# Effectiveness of Different Oral Health Interventions on Plaque and Gingivitis Incidence in Children Under Seven Years of Age: A Systematic Review and Meta-Analysis

**DOI:** 10.7759/cureus.67395

**Published:** 2024-08-21

**Authors:** Ashwini M Madawana, Mohamad Arif Awang Nawi, Akram Hassan

**Affiliations:** 1 Dentistry, School of Dental Sciences, Universiti Sains Malaysia, Kelantan, MYS; 2 Epidemiology and Public Health, School of Dental Sciences, Universiti Sains Malaysia, Kelantan, MYS

**Keywords:** gingivitis, plaque, dentistry, children, intervention, oral health

## Abstract

Oral health is crucial for young children, yet plaque and gingivitis pose significant challenges. This review evaluates oral health interventions for children under seven years to identify effective strategies. A systematic review was conducted across multiple databases using predefined criteria. Thirteen thousand five hundred records were identified, with 13 studies meeting the inclusion criteria. Various interventions were assessed, including tactile models, digital tools, fluoride varnish, and education programs. The meta-analysis showed heterogeneity in outcomes, with some interventions significantly reducing plaque and gingivitis. Tactile models and digital tools demonstrated effectiveness, particularly among children who were visually impaired and had congenital heart defects. However, fluoride varnish showed mixed results. School-based interventions and advanced toothbrushing technologies effectively reduce early childhood caries and plaque. Despite publication bias, a low risk of bias was observed across studies. The findings underscore the importance of tailored interventions and collaboration among stakeholders. Comprehensive oral health education, innovative digital tools, and multifaceted approaches are recommended to promote lifelong oral health habits. Further research is needed to standardize protocols and assess long-term effectiveness. Evidence-based interventions can significantly improve oral health outcomes for children under seven, laying the foundation for lifelong oral health.

## Introduction and background

Oral health is a critical health challenge, particularly in young children, where the foundation for lifelong habits is established. Plaque and gingivitis are common oral health issues that can lead to more severe dental problems if not correctly managed. According to a recent systematic review done by Kazeminia with a sample size of 80,405, the prevalence of dental caries in children's primary teeth was 46.2% (95% CI: 41.6-50.8%), while with a sample size of 1,454,871, the prevalence of dental caries in children's permanent teeth was 53.8% (95% CI: 50-57.5%) [1]. Various interventions have been developed and tested to improve oral hygiene and reduce the incidence of these conditions. This review aims to evaluate the effectiveness of different oral health interventions in reducing plaque and gingivitis among children under the age of seven. By analyzing various studies, we seek to identify the most effective strategies for improving oral health outcomes in this vulnerable age group.

Oral health is a fundamental aspect of overall well-being, particularly in young children, as it can significantly impact their growth, development, and quality of life. Dental plaque and gingivitis are prevalent oral health issues in children under seven years old, potentially leading to more severe dental problems if not adequately addressed. Effective prevention and management of these conditions are crucial to ensuring healthy oral hygiene habits from an early age. Plaque is a biofilm that forms on the teeth due to the accumulation of bacteria. If not removed regularly, it can lead to gingivitis, an inflammation of the gums characterized by redness, swelling, and bleeding. In young children, the development of plaque and gingivitis can be attributed to several factors, including poor oral hygiene practices, diet, and lack of awareness or education about oral health. Early intervention is critical in managing plaque and gingivitis in children. Establishing good oral hygiene habits, such as regular brushing and flossing, can prevent the onset of these conditions. Additionally, professional dental care and educational programs aimed at children and their caregivers play a significant role in promoting oral health.

Various oral health interventions have been implemented to reduce the incidence of plaque and gingivitis in young children. These interventions include educational programs that focus on increasing awareness and knowledge about oral hygiene practices among children and their parents or caregivers. They often involve interactive sessions, demonstrations, and the distribution of educational materials. Professional dental care such as regular dental check-ups, cleanings, and fluoride treatments provided by dental professionals are essential components of maintaining oral health in children. On the other hand, behavioral intervention techniques such as motivational interviewing and the use of rewards encourage consistent oral hygiene practices in children. In this modern era, the advent of technology has introduced digital tools and apps designed to engage children in oral hygiene routines through gamification and interactive learning. Despite the availability of various interventions, challenges remain in effectively reducing plaque and gingivitis incidence in young children. These challenges include ensuring accessibility to dental care, particularly in underserved communities, and addressing behavioral factors that influence oral hygiene practices. Additionally, the effectiveness of these interventions can vary based on individual and contextual factors, highlighting the need for tailored approaches.

## Review

Methodology 

A systematic review of relevant studies was conducted to assess the effectiveness of oral health interventions in children under seven years of age. The methodology involved a comprehensive search of multiple databases to identify studies that met predefined inclusion and exclusion criteria. The following steps outline the search strategy and criteria used in this review as seen in Table 1. 

**Table 1:** Search strategy This table shows databases, Boolean operators, search terms, and keywords used for this review. MEDLINE: Medical Literature Analysis and Retrieval System Online

Databases	PubMed, MEDLINE, Cochrane Library, Scopus, Google Scholar
Search terms and keywords	"oral health interventions" "plaque reduction" "gingivitis prevention" "Children under 7" "pediatric oral hygiene" "early childhood caries" "dental education" "fluoride varnish" "digital oral health applications" "toothbrushing programs"
Boolean operators	AND: to combine different aspects (e.g., "oral health interventions AND children under 7") OR: to include synonyms and related terms (e.g., "plaque reduction OR gingivitis prevention") NOT: to exclude irrelevant studies (e.g., "children under 7 NOT adolescents")

Inclusion Criteria

The inclusion criteria involved studies published in English, research involving children aged seven years and younger, including mixed dentition, studies examining interventions aimed at reducing plaque and gingivitis regardless of methods of plaque and gingivitis measurements, randomized controlled trials, clinical trials, longitudinal studies, studies with straightforward methodological design, and outcome measures.

Exclusion Criteria

The exclusion criteria involved studies involving participants older than seven years, research not focused on plaque or gingivitis as primary outcomes, reviews, meta-analyses, opinion pieces, studies lacking control groups, and non-peer-reviewed articles.

Data Extraction and Analysis

Data were extracted from each study on the following characteristics: authors, study design, objectives, sample size, intervention details, outcomes, and implications. All articles were extracted and stored in a separate EndNote tool (Clarivate, London, UK), and duplicates were removed. Studies were selected for inclusion by two different reviewers. Reviewer 1 evaluated titles and abstracts in duplicate, separately, while reviewer 2 approved studies based on the data and solved any disagreements on any included study. To determine whether the publications had the relevant data for the systematic review, the articles were fully examined by reviewers and were chosen for inclusion based on the following inclusion and exclusion criteria. A study characteristics table was created to summarize the findings and facilitate comparison across studies, as seen in Table 2. 

**Table 2:** Characters of the shortlisted studies This table shows reviewed studies on oral health interventions for children under seven that demonstrate various approaches to reducing plaque and gingivitis.

Author (s)	Method	Objective	Years	Sample size	Results	Implications
Chowdary et al. (2016) [2]	Clinical trial design	The study investigated the effect of tactile, braille text, and verbal oral hygiene.	6-16 years	Sample size N = 120; Intervention group = 80; Control group = 40	The study explored the impact of brushing techniques and good oral hygiene on the gingival and plaque indexes.	The study showed a reduction in gingivitis and dental plaque-impaired individuals.
Gautam et al. (2018) [3]	Prospective study	The study investigated the use of tactile, Braille, and audio models for the visually impaired.	6-16 years	Sample size N = 60; Intervention group N = 40; Control group = 20	The study observed reduced mean gingival and plaque scores for the intervention and control groups.	The combined use of Braille, tactile, and audio models improves oral health for visually impaired children.
Sivertsen et al. (2018) [4]	Prospective longitudinal study	The study determined the effectiveness of oral health education programs in children with congenital heart defects in controlling gingivitis.	2-5 years	Sample size was = 142; Intervention group; N = 75; Control group N = 67	The intervention group had reduced cases of gingivitis bleeding and dentine carriers compared to the control group upon using better oral hygiene.	The intensive oral health promotion program was articulated as an effective way of controlling gingivitis to avoid future risks of endocarditis diseases.
Shirmohammadi et al. (2022) [5]	Controlled clinical trial	The study assesses the effectiveness of smartphone programs in promoting oral health among children compared to regular oral health education.	2–6 years	Sample size N = 51; Intervention group N = 22; Control group N = 29	Children using smartphone programs significantly improved their gingival status more than the control group.	Smartphone programs for promoting oral health among children had a long-term impact on controlling gingivitis compared to standard oral health education programs.
Zotti et al. (2019) [6]	Randomized control trial	The study evaluated the effectiveness of apps in promoting children's oral hygiene against parental oral health education.	4–7 years	Sample size N = 100; Intervention group N = 50; Control group N = 50	There was vital oral hygiene and a low plaque index among children exposed to apps (intervention group) compared to those exposed to parental oral education (control group).	Children exposed to apps promoting oral education achieved higher compliance to control plaque infections and better oral health than those exposed to parental oral education.
Tadakamadla et al. (2022) [7]	A parallel-group, single-blinded, randomized controlled trial	The study investigated digital parenting intervention effect on oral health-related practices among children, practices of parents' health-related use while brushing their children's teeth, experiences with dental caries, and strategies for enhancing teeth brushing	2–6 years	Sample size N= 18; Intervention group N = 9; Control group N = 9	Parents using online oral-related practices, including toothbrushing and time spent on brushing, were associated with reduced dental caries and gingivitis among children compared to the control group.	The effectiveness of using online oral health practices can significantly improve the control of dental caries and oral health among children.
Rong et al. (2003) [8]	Clinical trial	The study explored the impact of implementing caries programs and oral health education for two years among three-year-old children.	1-3 years	Sample size N = 514; Intervention group N = 258; Control group N = 256	Children exposed to oral health education, including brushing their teeth twice a day using fluoridated toothpaste, had a high reduction in risks of gingivitis and plaque compared to those in the control group.	Developing oral education programs among preschool children can significantly promote effective oral habits, thus preventing the development of dental infections.
Tai et al. (2009) [9]	A cluster randomized controlled trial	The study investigated the effect of promoting oral health programs among schoolchildren over 3 years.	3–7 years	Sample size N = 1358; Intervention group N = 661; Control group N = 697	At three years, school children exposed to oral education (intervention group) achieved high scores in the prevention of plaques, gingivitis, and dental caries compared to the control group.	The study indicated that a school-based oral health program is an appropriate way of enhancing the reduction of plaque, gingivitis, and new caries and establishing effective behaviors of oral health practices among schoolchildren.
Muñoz‐Millán et al. (2018) [10]	A triple-blind randomized control trial	The study evaluated using biannual fluoride varnish in non-fluoridated remote areas to prevent early childhood caries.	2–3 years	Sample size N = 275; Intervention group N = 131; Control group N = 144	The reduction of caries and plaque risks was associated with children without fluoride varnish use compared to those using it.	The use of biannual fluoride varnish was articulated as not effective in controlling dental caries and plaques in schoolchildren living in fluoridated remote areas.
Oliveira et al. (2014). [11]	A randomized, examiner- and patient-blind, placebo-controlled, parallel-group design clinical trial	The study evaluated the effectiveness of using fluoride varnish to reduce adverse effects and caries incidents in children at six months.	1-4 years	Sample size N = 181; Intervention group N = 89; Control group = 92	The study found no significant difference between intervention and control groups in reducing the adverse effects and incidence of plaques and caries in children.	Using fluoride varnish had no impact on reducing caries and plaques among children.
Samuel et al. (2020). [12]	A double-blind, three parallel-arm clinical trial	The study examined the efficacy of school-based intervention in controlling early childhood caries in preschoolers.	3–5 years	Sample size N = 420' Intervention group = 210' Control group = 210	The study showed that the intervention group had an increased reduction in the risks of plaque and gingivitis compared to the control group.	The use of oral health education interventions, including daily supervision of teeth brushing and avoiding sugary snacks, prevents children from the risk of developing early childhood caries, plaque, and gingivitis.
Jeong et al. (2022) [13]	Randomized controlled clinical trial	The study compared the effectiveness of computer-based toothbrushing systems, including a smart toothbrush and smart mirror (STM) systems, against conventional toothbrushing in preventing plaque among children.	6–12 years	Sample size N = 42; Intervention group = 21; Control group = 12	The toothbrushing instruction (TBI) method of the smart toothbrush and smart mirror (STM) system (intervention) and convectional TBI (control) had no significant differences in reducing plaque risks.	The study found that despite having no significant differences between intervention and control, the intervention group was classified as the best alternative for children for toothbrushing to enhance plaque reduction.
Abdul Haq et al. (2023). [14]	Randomized controlled trial	The study investigated the effectiveness and acceptability of using digital applications in enhancing children's oral health evidence-based to control early childhood caries.	6-72 months	Sample size N = 43; Intervention N = 20; Control group N =23	Digital applications were considered an alternative tool for children to gain oral health knowledge compared to control (only verbal teaching)	The study highlighted that using digital applications was associated with increased evidence-based oral health in preventing early childhood caries among children, as recommended by parents.

Preferred Reporting Items for Systematic Reviews and Meta-Analyses (PRISMA) flow diagram

**Figure 1:**
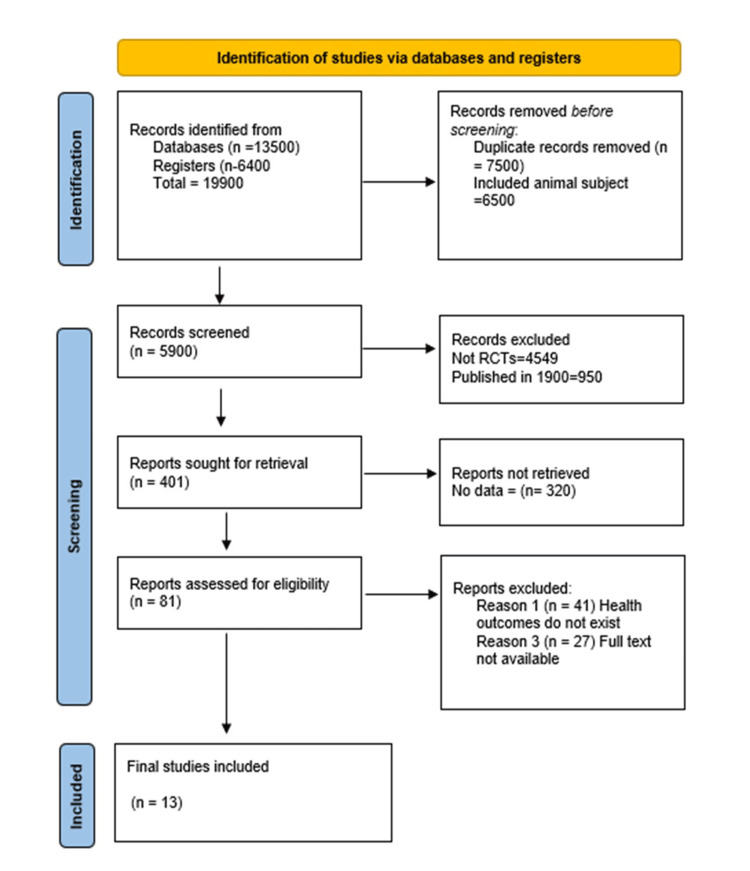
A PRISMA flowchart outlining the study selection process PRISMA: Preferred Reporting Items for Systematic Reviews and Meta-Analyses

The data extraction process followed the PRISMA guidelines and the above search strategy as seen in Figure 1. From the mentioned databases, 13,500 records were identified together with 6,400 registers. A total of 19,900 studies were identified from both registers and databases. Nevertheless, 7,500 were duplicates, and 6,500 were grey literature, conference abstracts, and books; therefore, 14,000 records were excluded; 5,900 records underwent the screening procedure, but 4,549 were not quantitative research and, therefore, excluded. Also, 950 records were dated in the 1900s and excluded from this research. Only 401 records were sought for retrieval, but 320 had no data. Eighty-one records were assessed for eligibility, 14 lacked human health outcomes, and 27 were not in full text. Only 13 records met the inclusion criteria.

Results 

The reviewed studies on oral health interventions for children under seven demonstrate various approaches to reducing plaque and gingivitis, highlighting the effectiveness of innovative educational techniques, digital tools, and fluoride applications, as seen in Table 2. Chowdary and Gautam supported using tactile, Braille, and audio models, demonstrating reductions in gingivitis and plaque scores, emphasizing the importance of accessible oral health education for visually impaired children. In this study, children's gingival and plaque scores decreased in every group. Group II had the largest decrease in gingival scores (84%), whereas Group III had the most percentage reduction in plaque scores (70.6%) [2-3]. Sivertsen conducted a prospective longitudinal study on children with congenital heart defects, revealing that intensive oral health promotion programs can effectively reduce gingivitis and prevent future health complications. In this study, children (d1-5mft) with caries in the intervention group had fewer caries-affected teeth than those in the control group (p = 0.06) [4].

Shirmohammadi and Zotti explored the impact of digital tools, such as smartphone programs and educational apps, on children's oral health [5-6]. Shirmohammadi concludes that a better modified gingival index (MGI) was found in the application intervention group at the three-month follow-up (p<0.001) [5]. In Zotti's study, one might suggest that the different characteristics of the drop of plaque index (PI) in two groups in the first three months could be attributable to the use of application assistance by the study group's (SG) patients. In the SG, a decrease of PI was detected in t1, and its levels were constant over time. In actuality, the PI in the SG decreased from 2,45 to 1,56, but the PI in the control group (CG) decreased less sharply from 2.3 to 2. It's possible that the SG patients' downloaded apps promoted more caution when carrying out oral hygiene practices [6]. Both studies showed significant improvements in gingival health and plaque control, with Zotti et al. highlighting higher compliance rates in children using apps compared to those receiving parental oral education [6].

Further supporting the efficacy of digital interventions, Tadakamadla demonstrated that digital parenting programs improved oral health practices, reducing dental caries and gingivitis [7]. Rong and Tai focused on school-based and community-based oral health education programs, finding substantial reductions in plaque, gingivitis, and dental caries [8,9]. In Rong's study, better MGI was found in the application intervention group at the three-month follow-up (p<0.001) [8]. On the other hand, according to Tai, after three years, children from the intervention group had significantly higher scores than those from the control group for restorations received, sealants applied, and untreated dental caries (P<0.01) [9]. These studies underscore the long-term benefits of early and consistent oral health education.

In contrast, with differing outcomes, Muñoz-Millán and Oliveira examined fluoride varnish. Muñoz-Millán et al. reported no significant reduction in dental caries and plaque among children in non-fluoridated areas [10-11]. At the same time, Oliveira et al. found no notable impact of fluoride varnish on reducing caries and plaques. These findings suggest that fluoride varnish alone may not be sufficient for effective oral health management [11].

Samuel and Jeong evaluated various school-based interventions and advanced toothbrushing technologies [12-13]. Samuel demonstrated that supervised brushing and dietary modifications significantly reduced early childhood caries, plaque, and gingivitis. Jeong found no significant difference between smart toothbrush systems and conventional methods, although smart technologies were favored for enhancing brushing practices. On the other hand, Abdul Haq emphasized the use of digital applications was associated with increased evidence-based oral health in preventing early childhood caries among children, as recommended by parents [14]. In this study, the approximate plaque index (API) was significantly better at the follow-up in the test arm (p = 0.01), with no differences in the control arm (p = 0.72).

The diverse methodologies and interventions reviewed highlight the importance of tailored educational techniques, digital tools, and comprehensive oral health programs in effectively reducing plaque and gingivitis among young children. The mixed results on fluoride varnish suggest the need for multifaceted approaches to oral health promotion.

Meta-analysis 

The meta-analysis section quantifies the data to provide visualized results. The analysis applies the Mantel-Haenszel (M-H) technique and random effect to calculate the overall effect of the analysis. Table 2 shows the synthesized data for meta-analysis. Table 3 is the summary of critical quantitative data for meta-analysis. The table shows the total sample sizes, intervention, and control sample sizes with the age of the participants.

**Table 3:** Synthesized data

Author(s)	Total sample size	Intervention sample size	Control sample size	Age range
Chowdary et al. (2016) [2]	120	80	40	6-16 years
Gautam et al. (2018) [3]	60	40	20	6-16 years
Sivertsen et al. (2018) [4]	142	75	67	2-5 years
Shirmohammadi et al.(2022) [5]	51	22	29	2-6 years
Zotti et al. (2019) [6]	100	50	50	4-7 years
Tadakamadla et al. (2022) [7]	18	9	9	2-6 years
Rong et al. (2003) [8]	514	258	256	1-3 years
Tai et al. (2009) [9]	1358	661	697	3-7 years
Muñoz‐Millán et al. (2018) [10]	275	131	144	2-3 years
Oliveira et al. (2014). [11]	181	89	92	1-4 years
Samuel et al. (2020). [12]	420	210	210	3-5 years
Jeong et al. (2022) [13]	42	21	21	6-12 years
Abdul Haq et al. (2023). [14]	43	20	23	6-72 months

Forest plot 

**Figure 2:**
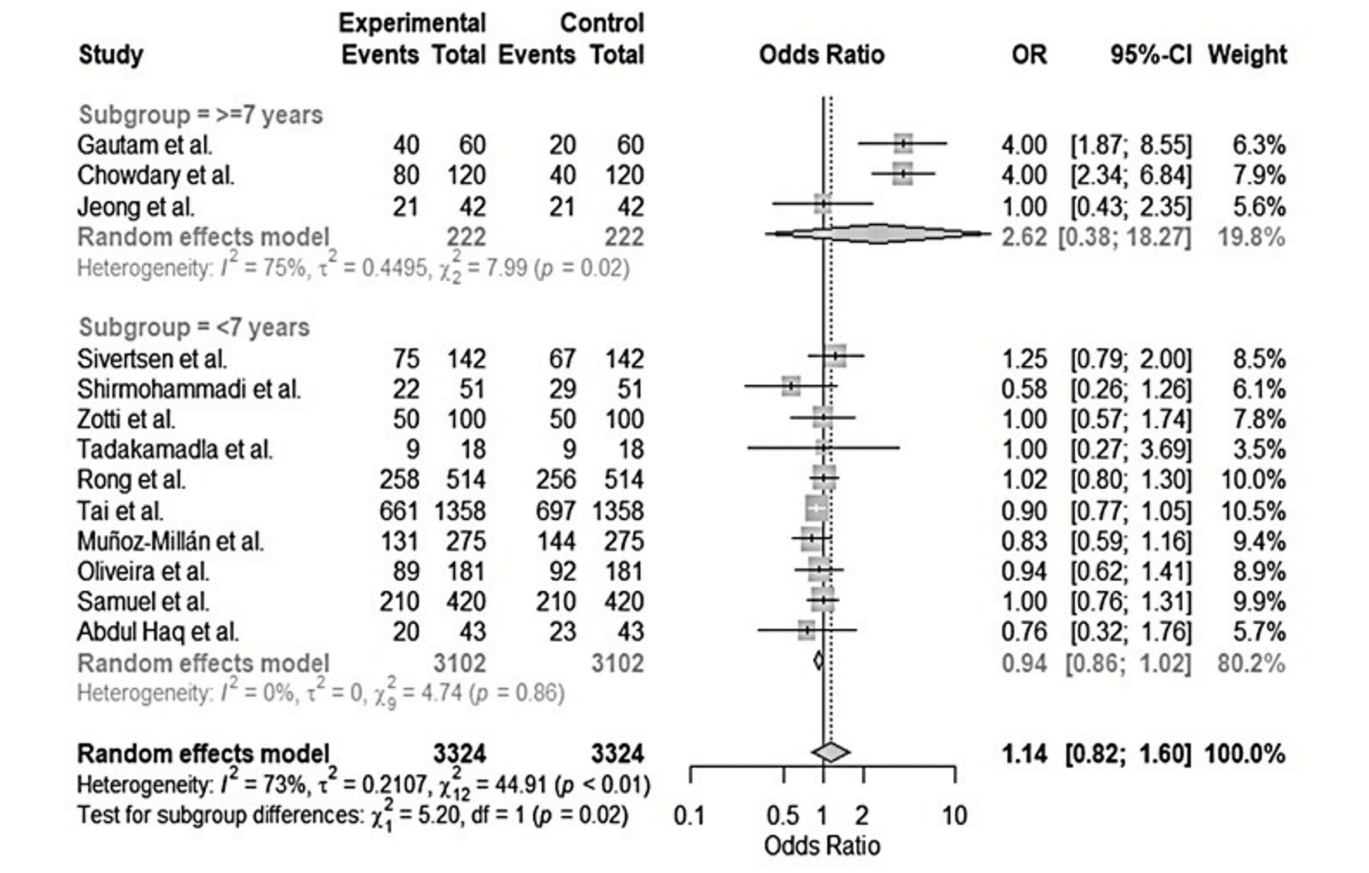
The forest plot provides the overall effect of each study in establishing the effectiveness of the different oral health interventions on plaque and gingivitis incidence in children under seven years. References: [2-14]

The forest plot in Figure 2 visualizes the Table 2 data to provide the overall effect of each study in establishing the effectiveness of the different oral health interventions on plaque and gingivitis incidence in children under seven years. Subgroup 1 shows studies investigating the oral health of children aged 0 to more than seven years, while subgroup 2 had a majority of children who were seven years and below. Subgroup 1 data was substantially heterogeneous, although the results showed significant changes in the plaque and gingivitis incidences for the children in the specified age group. In this respect, Chowdary, Gautam, and Jeong’s interventions significantly promoted oral health among the children, as seen in the odds ratio values [2,3,13]. Subgroup 2 was not heterogeneous, meaning the results reflect this study's objectives. However, only studies by Sivertsen and Rong show significant positive changes in oral health interventions [4,8]. Other studies under subgroup 2 were insignificant due to the lower values in the odds ratio, which may have been influenced by sample size and study duration.

Funnel plot 

**Figure 3:**
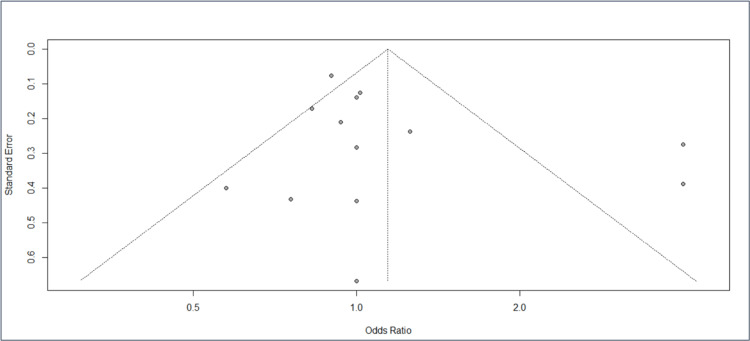
The funnel plot indicates the presence of publication bias in all of the assessed studies References: [2-13]

The funnel plot in Figure 3 indicates the presence of publication bias. The included studies have produced a symmetrical funnel plot, which indicates a lack of publication bias among the studies.

Risk of bias 

**Figure 4:**
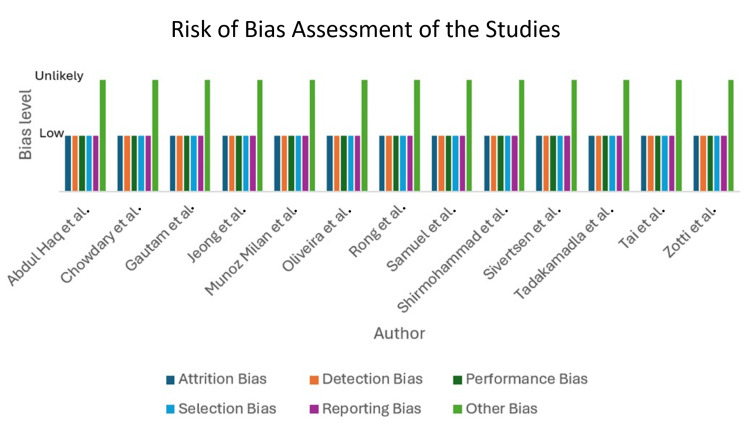
Risk of bias assessment of each included study In this respect, the main elements were attrition bias, detection bias, performance bias, selection bias, and other biases. References: [2-14]

It was imperative to assess the risk of bias for each study included. In this respect, the main elements were attrition bias, detection bias, performance bias, selection bias, and other biases. The results observed low biases unless the other biases were detected as unlikely. An elaborate risk of bias graph is shown in Figure 4.

Discussion 

The oral health interventions reviewed in the meta-analysis exhibit varying effectiveness in reducing plaque and gingivitis among children under seven. Each intervention has its strengths and weaknesses, contributing to this population's overall landscape of oral health promotion. Chowdary and Gautam demonstrated the effectiveness of tactile, Braille, and audio models in reducing plaque and gingivitis among visually impaired children [2-3]. These interventions are commendable for their innovative approach to making oral health education accessible. These models not only educate but also empower visually impaired children to maintain better oral hygiene independently. The effectiveness of these interventions is notable as they significantly reduce plaque and gingivitis in this specific population. However, their broad applicability might be limited, as these interventions are tailored to address the unique needs of visually impaired children.

Sivertsen highlighted the success of intensive oral health promotion programs, particularly in children with congenital heart defects [4]. These programs are crucial, as children with congenital heart defects are at a higher risk for developing oral health problems, which can lead to severe complications. The strength of these interventions lies in their targeted approach, addressing the specific health conditions and preventing future complications. This specificity ensures that the oral health needs of these children are met comprehensively. Similarly, Shirmohammadi and Zotti showcased the potential of digital tools to improve oral health outcomes, offering scalability and convenience [5-6]. Digital platforms and applications provide scalable solutions that can reach a broad audience, offering convenience and interactive learning experiences. These tools are especially beneficial in an increasingly digital world, where technology can enhance traditional health education methods. However, there are challenges associated with the reliance on technology.

Access to digital tools can be limited by socioeconomic factors, potentially excluding certain populations. Moreover, there can be barriers related to digital literacy, which must be addressed to ensure the effectiveness of these interventions. Studies such as those by Chowdary, Gautam, and Sivertsen et al. demonstrate the potential of innovative strategies [2-4]. At the same time, research by Muñoz-Millán and Oliveira underscores the need for multifaceted approaches beyond fluoride varnish applications alone [10-11]. These findings highlight the need for comprehensive interventions that combine educational techniques, digital tools, and intensive programs. Such multifaceted approaches are more likely to yield sustainable improvements in oral health by addressing various determinants of health and ensuring broader accessibility and engagement.

The reviewed interventions in the meta-analysis present a spectrum of strategies to reduce plaque and gingivitis among children under seven. Each approach, from innovative tactile and audio models for visually impaired children to intensive programs for those with specific health conditions and scalable digital tools, contributes uniquely to the landscape of oral health promotion. The integration of these diverse methodologies can lead to more effective and inclusive oral health strategies, ultimately improving outcomes for children in this age group. The need for multifaceted approaches is evident, as a combination of interventions can address the varied and complex factors influencing oral health.

However, since the strategies were introduced in children, the implementation, adherence, and compliance could be a daunting task in applying the above-mentioned strategies. It should also be taken into consideration that not all the strategies mentioned are accessible to children all around the globe. Hence, a more affordable and accessible alternative should be researched and studied in the future.

## Conclusions

To significantly improve oral health outcomes for young children, a combination of immediate and long-term strategies must be employed. In the short term, there is an urgent need to expand comprehensive oral health education programs. These should be integrated into both school-based and community-based settings, where they can effectively reach children and their families. These programs should not only emphasize the importance of oral hygiene but also incorporate supervised brushing sessions and dietary education, teaching children the impact of nutrition on oral health. Additionally, leveraging innovative digital tools, such as smartphone apps and digital parenting programs, can enhance engagement. These tools can provide reminders, educational content, and interactive activities that motivate both children and parents to maintain good oral health practices at home.

Simultaneously, fostering collaboration among key stakeholders like researchers, healthcare providers, educators, and policymakers is critical to ensuring that these strategies are evidence-based and tailored to meet the needs of diverse populations. Such collaboration can help in creating standardized protocols for oral health interventions, which is a crucial long-term goal. Standardization ensures that interventions are consistently applied across different settings and populations, enhancing their effectiveness and reliability.

Long-term efforts should also focus on conducting longitudinal studies to assess the lasting impact of these interventions on children's oral health and overall well-being. Understanding the long-term outcomes will help in refining and improving strategies over time. Addressing potential biases in current research methodologies is equally important, as it ensures that the findings are robust and applicable across different demographic groups.

Finally, it is essential to broaden the scope of oral health interventions to encompass a multifactorial approach. This includes not only traditional methods like fluoride varnish applications but also dietary modifications, regular dental check-ups, and education on the importance of reducing sugar intake. By combining these immediate actions with a commitment to long-term goals, stakeholders can make substantial progress in improving oral health outcomes for children, laying the foundation for lifelong healthy habits.
